# Canine atopic dermatitis: detailed guidelines for diagnosis and allergen identification

**DOI:** 10.1186/s12917-015-0515-5

**Published:** 2015-08-11

**Authors:** Patrick Hensel, Domenico Santoro, Claude Favrot, Peter Hill, Craig Griffin

**Affiliations:** Tierdermatologie Basel, Emil Frey-Strasse 127, Münchenstein, Switzerland; Department of Small Animal Clinical Sciences, College of Veterinary Medicine, University of Florida, Gainesville, FL USA; Vetsuisse Faculty University of Zurich, Clinic of Small Animal Internal Medicine, Zurich, Switzerland; Companion Animal Health Centre, School of Animal and Veterinary Sciences, University of Adelaide, Roseworthy, SA 5371 Australia; Animal Dermatology Clinic, San Diego, CA USA

## Abstract

**Background:**

Canine atopic dermatitis (AD) is a common, genetically predisposed, inflammatory and pruritic skin disease. The variation in clinical presentations, due to genetic factors, extent of the lesions, stage of the disease, secondary infections, as well as resemblance to other non-atopic related skin diseases, can complicate a diagnosis of canine AD. A sub-group of the International Committee for Allergic Diseases in Animals (ICADA) was tasked with the development of a set of practical guidelines that can be used to assist practitioners and researchers in the diagnosis of canine AD. Online citation databases and abstracts from international meetings were searched for publications related to the topic, and combined with expert opinion where necessary. The final set of guidelines was approved by the entire ICADA committee.

**Results:**

A total of 81 publications relevant for this review were identified. The guidelines generated focus on three aspects of the diagnostic approach:

1. Ruling out of other skin conditions with clinical signs resembling, or overlapping with canine AD.
2. Detailed interpretation of the historical and clinical features of patients affected by canine AD.
3. Allergy testing by intradermal versus allergen-specific IgE serum testing.

**Conclusions:**

The diagnosis of canine AD is based on meeting clinical criteria and ruling out other possible causes with similar clinical signs. Flea combing, skin scraping and cytology should be performed, where necessary, as part of a thorough work-up. Elimination diet trials are required for patients with perennial pruritus and/or concurrent gastrointestinal signs. Once a clinical diagnosis of canine AD is made, allergy testing can be performed to identify potential causative allergens for allergen-specific immunotherapy.

## Background

Canine Atopic Dermatitis (AD) has been defined as a genetically predisposed inflammatory and pruritic allergic skin disease with characteristic clinical features. It is associated most commonly with IgE antibodies to environmental allergens [1]. Although this definition encompasses many aspects of the pathogenesis and clinical aspects of the condition, it is important to remember that this disease has no pathognomonic clinical signs that permit a definitive diagnosis to be made upon initial owner interview and clinical examination [2]. This is due to the diversity of the clinical presentation, which may depend on genetic factors (breed-associated phenotypes) [3, 4], extent of the lesions (localised versus generalised), stage of the disease (acute versus chronic), and the presence of secondary microbial infections or other flare factors. Furthermore, some aspects of the disease can resemble other skin conditions that are not related to canine AD. For the above-mentioned reasons, the definitive diagnosis of canine AD can be difficult.

A sub-group of the International Committee for Allergic Diseases in Animals (ICADA) developed, based on extensive searches in online citation databases and abstracts from international meetings, a set of practical guidelines that can be used to assist practitioners and researchers in the diagnosis of canine AD.

These guidelines provide an overview of the diagnosis of canine AD that involves three distinct, but complementary, approaches. These are:

1. Ruling out of other skin conditions with clinical signs that can resemble, or overlap with canine AD. This is traditionally referred to as “the work-up”.
2. Detailed interpretation of the historical and clinical features of the condition. A new tool to assist with interpretation of these findings is the application of clinical criteria known as “Favrot’s criteria” [5].
3. Assessment of skin reactivity by IntraDermal Testing (IDT) or detection of IgE by Allergen-Specific IgE Serology (ASIS) testing. This is traditionally referred to as “allergy testing”.

Use of any one of these approaches in isolation can result in misdiagnosis, so it is important not to rely on any of them as a sole diagnostic principle.

### Ruling out of other skin conditions with clinical signs that can resemble, or overlap with, canine AD

The evaluation of a pruritic dog requires a step-by-step thought-process and approach that should lead to a definitive diagnosis. The differential diagnoses and role of complicating factors (Table 1) need to be narrowed down using information derived from the history, the findings on physical examination, diagnostic tests (where necessary), and response to treatment. Basic sampling methods and diagnostic tests, which may be required to rule out most of the common differentials are flea combing, skin scraping, hair plucking and cytological examination of skin and ear samples. Depending on the complexity of the case, the following steps may be performed over a series of visits, or all at once.

**Table 1:** Important differential diagnoses for pruritic skin diseases in dogs

Ectoparasitic skin diseases	Fleas
	Scabies (*Sarcoptes scabiei*)
	Demodicosis
	Cheyletiellosis
	Pediculosis
	Otoacariasis (*Otodectes cynotis*)
	Trombiculiasis
	Nasal mites (*Pneumonyssus caninum*)
Microbial skin infections	Staphylococcal pyoderma
	Malassezia dermatitis
Allergic skin diseases	Flea allergy dermatitis
	Atopic dermatitis
	Food intolerance/allergy
	Insect bite hypersensitivity
	Contact dermatitis
Neoplastic disease	Cutaneous lymphoma

### Step 1 – Consider the possibility of fleas

**Fig. 1:**
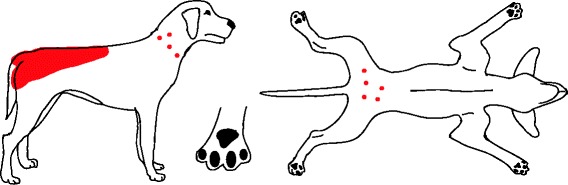
Distribution of skin lesions and pruritus associated with FAD. Acute lesions: Erythematous macules, papules, crusted papules, hot spots. Chronic lesions: Self-induced alopecia, lichenification, and hyperpigmentation

While the clinical signs in a dog with flea infestation are variable, the location of skin lesions and pruritus associated with flea allergy dermatitis (FAD) are most commonly found at the lumbosacral area, tail base and caudomedial thighs (Fig. 1) [6]. A flea infestation is associated with increased flea counts, whereas in dogs with FAD this may not be the case. In addition, clinicians must be aware that many atopic dogs may suffer from concurrent FAD, which may complicate the clinical diagnosis.

To exclude FAD or flea infestation as a possible cause of pruritus in a particular case, clinicians should apply the following guidelines:

- The prevalence of fleas and associated hypersensitivities depends on the geographical area in which the animal lives. Fleas can be a perennial problem in subtropical and tropical climate zones, seasonal in more tempered climate zones and practically non-existent in arid, high elevation, or cold climates [7, 8]. Even if fleas are considered to be absent from a particular area, clinicians should consider any recent travel history to flea endemic areas or contact with animals from such areas.
- In dogs with pruritus and/or lesions in areas of the body that are not primarily affected by fleas (e.g., the paws or ear canals), FAD may not be the sole cause of pruritus.
- Clinicians should check all pruritic dogs for fleas or flea faeces on direct examination or brushing the hair coat (flea combing). To exclude FAD when fleas or flea faeces cannot be found, an effective flea control program should be initiated. Clinicians should be aware that none of the current flea preventatives have an effective repellent effect, and that the fleas in the pupal stage can survive up to 174 days [9]. Based on duration of survival it is recommended to maintain consistent flea prevention in flea endemic areas. It is also advised that fast-acting systemic adulticides are used as these may be more effective at reducing pruritus quickly compared to other topically applied flea preventatives [10].
- Cases that are being entered into a study of canine AD should undergo effective flea control prior to study enrollment. Because the duration of flea control, prior to study inclusion, may influence the outcome of such trials, a recent study suggests that dogs should be on flea prevention for at least 3 months prior to study enrollment [11]. In addition, all other dogs and cats in the household need to be on effective flea control as well.

### Step 2 – Consider the possibility of other ectoparasites

**Fig. 2:**
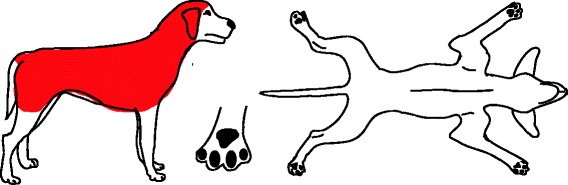
Distribution of skin lesions and pruritus associated with Lice/Cheyletiella. Lice: No visible lesions, or mild scaling and excoriation. Cheyletiella: Marked dorsal seborrhea

**Fig. 3:**
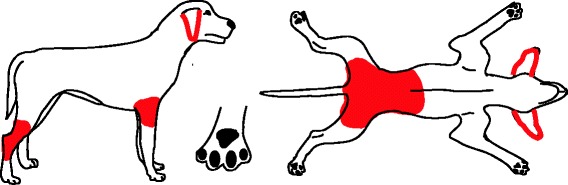
Distribution of skin lesions and pruritus associated with sarcoptic mange. Lesions include papular eruption, erythema, scaling, excoriations

**Fig. 4:**
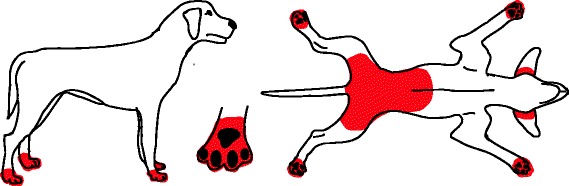
Distribution of skin lesions and pruritus associated with trombiculiasis. Lesions usually manifest as eruption

**Fig. 5:**
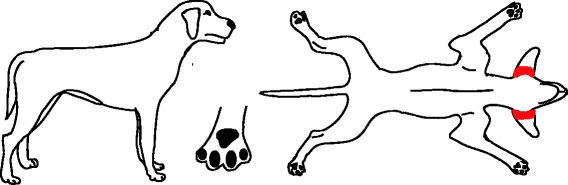
Distribution of skin lesions and pruritus associated with otoacariasis. Lesions include erythema, dark-brown, coffee-ground like discharge

**Fig. 6:**
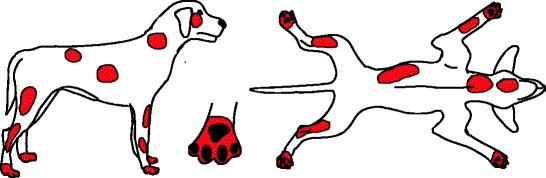
Distribution of skin lesions and pruritus associated with demodicosis. Lesions include focal, multi-focal or generalised alopecia, scaling, erythema, follicular casts, comedones, Furunculosis

Besides fleas, other ectoparasites may be associated with pruritus (e.g., sarcoptic mange, cheyletiellosis, pediculosis, trombiculiasis, otoacariasis) or can be found as a concurrent disease (e.g., demodicosis). Although the majority of these parasites favour specific body areas (Figs. 2, 3, 4, 5 and 6), they can be difficult to distinguish clinically.

Prior to an allergy investigation, every attempt should be made to rule out potential ectoparasitic skin diseases. Various sampling methods such as skin scraping, hair combing, hair plucking, ear swabbing, and acetate tape impressions can be used to collect specimens. For the identification of these parasites a microscopic examination with a low-power objective (4× or 10×) and low light intensity should be used [12]. The following list indicates which sampling methods are effectively used for various ectoparasites:

- *Sarcoptes scabiei var. canis*: Microscopic examination of multiple superficial skin scrapings, and, where available, blood serum for serology testing (indirect Enzyme-Linked ImmunoSorbent Assay (ELISA) [13, 14]. Sarcoptes mites can occasionally be found on skin biopsies and fecal flotation [15].
- *Demodex spp.*: Microscopic examination of multiple deep skin scrapings and acetate tape impressions of “squeezed” skin, and hair pluckings [16, 17]. Usually *Demodex* mites are easy to find if multiple affected body areas are sampled. However, sampling infected feet or in breeds with thick skin (e.g., shar peis) may not always be effective and skin biopsies may sometimes be required [18].
- *Cheyletiella spp.*, *Trombicula spp.* (chiggers)*,* and lice: Microscopic examination of coat brushings, acetate tape impressions and superficial skin scrapings [15]. *Cheyletiella spp*. and lice also produce eggs, which are attached to hair shafts and can be identified by trichography.
- *Otodectes cynotis*: Microscopic examination of aural discharge. The discharge often appears dark brown-black and crumbly (coffee ground-like) and the mites are white, very mobile and light shy. Occasionally ear mites can be found on superficial skin scrapings at other body sites [19].

*Sarcoptes scabiei var. canis* and *Cheyletiella spp*. can be difficult to find [15, 20]. For this reason a response to an antiparasitic trial treatment (e.g., selamectin, moxidectin, ivermectin, amitraz, lime sulfur) may be necessary to rule out these parasites. A positive pinnal pedal reflex has been associated with Sarcoptes and justifies trial therapy [21]. Especially in the light that Sarcoptic mites are able to cross-react with house dust mites (HDM) in allergy testing, a trial treatment in very pruritic patients is strongly recommended [22, 23].

### Step 3 – Consider the possibility of Staphylococcal infection and *Malassezia* overgrowth

#### Pyoderma

Bacterial skin infections caused by *Staphylococcus pseudintermedius* (SP) are common in dogs with AD. The typical lesions of superficial pyoderma, such as papulo-pustular eruption and epidermal collarettes, are often distinctive enough to make a clinical diagnosis on gross appearance alone. However, the initial diagnosis should be confirmed by examining cytological samples, stained with Diff-Quik®, taken from the skin by impression smears or acetate tape impressions [12, 24]. Samples from pricked pustules will most likely yield definitive results, while samples from papules and epidermal collarettes may be less rewarding. Aerobic bacterial culture and sensitivity testing is not indicated in every case, but if particular conditions are fulfilled (e.g., previous history of antibiotic treatment, initial appropriate antibacterial treatment has not been effective, high prevalence of meticillin-resistant SP in the area, etc.), a bacterial culture with antibiogram should be performed [25]. Bacterial cultures can be performed while the dog is currently being treated with systemic antibiotics [26].

Staphylococcal pyoderma is in most cases a secondary problem associated with underlying pruritic and non-pruritic diseases such as canine AD, but also other allergies as well as endocrinopathies. The pyoderma often causes a change in the overall level or distribution pattern of the pruritus. In these cases, eliminating the pyoderma will determine if the primary disease is itself pruritic, and what its severity and distribution pattern may be. In addition to typical pyoderma lesions, dogs with AD can develop bacterial overgrowth that can complicate other lesion types. Hence, it is wise to sample a variety of lesions to characterise the extent of bacterial involvement and manage the infection appropriately. This should certainly be done whenever cases are poorly responsive to “anti-allergy” therapies, or if studies on canine AD are being performed.

#### Malassezia dermatitis

**Fig. 7:**
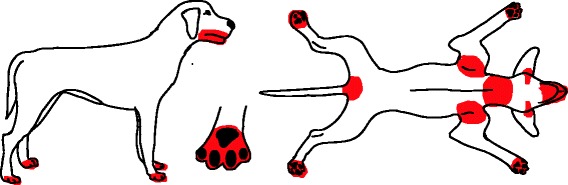
Distribution of skin lesions and pruritus associated with *Malassezia* dermatitis. Lesions include erythema, yellowish or brownish greasy scale, hyperpigmentation

The most effective diagnostic test for the identification of *Malassezia* organisms is skin cytology from affected areas such as skin folds, areas with lichenification and oily seborrhea (Fig. 7) [12, 24]. *Malassezia pachydermatis* is a budding yeast organism (3–5 μm in diameter) with a characteristic oval, peanut or “Russian doll” shape, allowing easy identification. In general, clinical signs associated with the cytological presence of yeasts reflect a yeast overgrowth or infection. However, in dogs with *Malassezia* hypersensitivity, few organisms may elicit pruritus and associated skin lesions. For this reason a diagnosis of *Malassezia* dermatitis should be based on the clinical and cytological findings and confirmed by a response to antifungal therapy [27]. Fungal culturing can be performed as well, but is not used routinely for the diagnosis of *Malassezia* dermatitis, because false negative culture results have been reported [28, 29]. Therefore, in studies of canine AD, the presence of any number of *Malassezia* organisms should warrant a trial therapy to determine what role, if any, low numbers of *Malassezia* are playing in causing the dog’s pruritus.

### Step 4 – Consider the role of cutaneous adverse food reaction (CAFR)

Food related pruritus can be caused by two different mechanisms, one a non-immune mediated reaction (food intolerance), the other immune mediated which includes IgE-mediated hypersensitivity (food allergy) [30]. Because reactions to food components can present clinically as canine AD, or serve as a flare factor in canine AD, dogs with CAFR may be indistinguishable clinically from canine AD [31–33]. The presence of gastrointestinal signs, such as diarrhoea, vomiting, tenesmus, soft stools, flatulence, and increased number of bowel movements is more typically seen with food-induced canine AD [5, 33]. In any canine AD case that has year-round clinical signs, CAFR can only be ruled out by effective strict elimination diet trials, since accurate diagnostic commercial tests are not currently available. This is especially important in trials evaluating drugs for the treatment of canine AD since food-induced AD may not respond well to those drugs, as shown for corticosteroids [5]. Unfortunately, there are no diets that have been shown to be effective in all cases of CAFR. Therefore in some cases, especially when gastrointestinal signs are present, multiple different diet trials may be needed until a sufficient control of the clinical signs has been achieved.

Ideally an elimination diet trial should be performed with a diet to which ingredients the dog has never been exposed before. Unfortunately, most commercially available diets contain a wide range of ingredients and by-products, making the selection of an appropriate diet difficult. Most over the counter diets as well as some prescription elimination diets may be contaminated with traces of other food components [34, 35]. Although hydrolysed diets are offered as an alternative option, the protein source is based on either chicken or soy. For this reason some dogs allergic to chicken and/or soy may not respond to such diets [36]. The most common food allergens in dogs are: beef, dairy, chicken products and wheat, and to a lower degree soy, lamb, pork, fish, and corn [37].

A diet trial is performed by instituting a strict trial with a diet containing commercial or home-cooked novel (e.g., rabbit, kangaroo, venison, horse, etc.) or hydrolysed protein ingredients. The use of these novel proteins is becoming more problematic because several of these novel proteins are now available in over the counter commercial diets. A study in humans has also shown that venison does cross-react in vitro with bovine IgG [38], while another study reported that up to 85 % of food allergic dogs may adversely react to venison [39]. Any strict elimination diet trial should be fed exclusively for a minimum of 8 weeks to achieve complete clinical remission in most cases [40]. If the condition improves, the diet should be continued to determine if there is complete or only partial control of the clinical signs. If a dog is not responding to a commercial elimination diet a second attempt with a home-cooked diet should be performed [34]. Home-cooked diets are considered the most limited ingredient diets if done properly. All diet trials should be continued until the veterinarian examines the dog. This is important as some owners may not recognize a partial response or be aware of lesions still present when a dog appears to have improved. Dietary involvement is confirmed if there is a relapse of clinical disease when the original diet is re-introduced. Clinicians should be aware that poor owner/patient compliance is a common problem. Typical pitfalls during a diet trial are: feeding table food, raw hides, treats, “hiding” medication in food, using flavoured tooth paste, giving medication in gelatine capsules, using flavoured drugs (e.g., NSAIDs, antibiotics, chewable heartworm or flea preventative), and dogs eating other animals’ faeces. Clients need to realize that very small amounts of other foods or food additives ingested, even intermittently, can prevent a favourable response [41]. Crumbs on the floor and even licking another pet’s empty bowl may result in a poor outcome. The client’s job is to make sure the dog ingests nothing but the prescribed diet and water.

Once steps 1–4 of the diagnostic work-up has been completed, a clinical diagnosis of canine AD should be considered if the pruritus is still present.

### Detailed interpretation of the historical and clinical features of canine AD

**Fig. 8:**
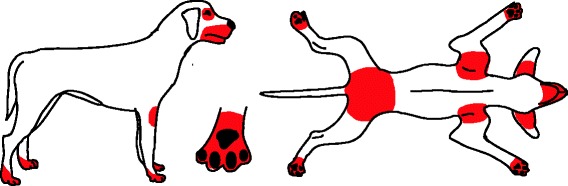
Common distribution of clinical lesions and pruritus associated with canine AD and food allergy

**Fig. 9:**
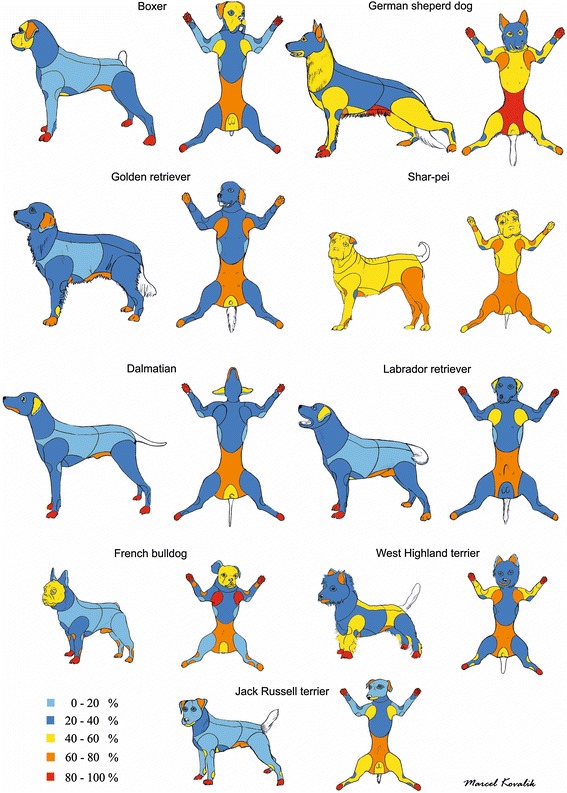
Silhouettes of atopic boxers, German shepherd dog, golden retrievers, shar peis, Dalmations, Labradors retriever, French bulldogs, West Highland white terriers and Jack Russell terriers (in this order). Each colour corresponds to the percentage of affected animals (Reproduced with permission from Veterinary Dermatology)

**Fig. 10:**
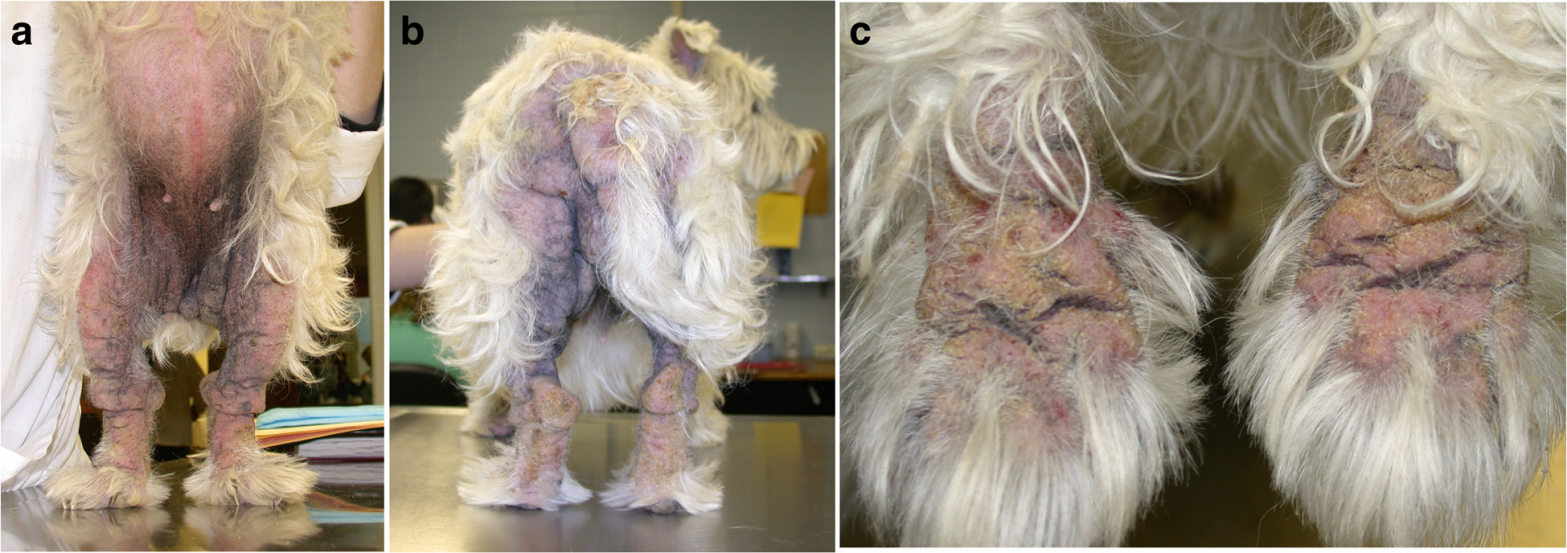
**a**, **b**, **c** Typical distribution of secondary skin lesions in a West Highland white terrier

The initial clinical feature of canine AD is pruritus, which can include scratching, rubbing, chewing, excessive grooming or licking, scooting, and/or head shaking. Depending on the allergens involved, the pruritus may be seasonal (e.g., pollen) or non-seasonal (e.g., dust mites, food) [42]. At the beginning the pruritus may be alesional or associated with primary skin lesions such as erythema and occasionally papules (Table 2) [43, 44]. The face, concave aspect of the ear pinnae, ventrum, axillae, inguinal area, perineal area and distal extremities are most commonly affected in canine AD (Fig. 8) [43], but breed-associated variations of body sites affected by canine AD have been identified (Table 3, Fig. 9) [3]. In more chronic stages secondary skin lesions (Table 2) will occur due to self-trauma, chronic inflammation and secondary infections. Typical secondary skin lesions are excoriations, alopecia, lichenification, hyperpigmentation, crusting, and seborrhea (Fig. 10a-c).

**Table 2:** Key dermatologic features for canine pruritic skin diseases

Alesional Pruritus	May be seen in the early stages of allergy or when seasonal disease begins. This finding of pruritus in areas with no lesions can occur in canine AD cases at any point in the disease process, especially in cases that have recurrences or come out of remission.
Primary skin lesions	
Erythema	Can be seen with most of the above differentials, but lice and Cheyletiella do not usually cause erythema. Demodicosis is highly variable – the skin may or may not appear to be inflamed.
Papules	Seen with flea bites, scabies, Trombiculiasis, insect bite hypersensitivity, staphylococcal pyoderma, atopic dermatitis, cutaneous adverse food reaction, and contact dermatitis. Dogs with AD may have small non-crusted papules unless there are concurrent diseases.
Pustules	Most commonly associated with staphylococcal pyoderma
Secondary skin lesions	
Epidermal collarettes	Most commonly associated with staphylococcal pyoderma
Crusting	Most commonly associated with secondary infections and excoriations
Salivary staining	Indicates excessive licking and often associated with Malassezia
Excoriations	Self-induced trauma from scratching due to severe pruritus
Alopecia	May be due to self-trauma or folliculitis (superficial pyoderma, demodicosis, and dermatophytosis)
Lichenification	Indicates chronic pruritus, inflammation and commonly associated with secondary infections
Hyperpigmentation	Indicates chronic pruritus. Allergies and Malassezia are the most common causes and result dark discoloration of the skin. Blue-grey pigmentation is seen with demodicosis in some cases.

**Table 3:** Additional body sites involved in canine AD in certain breeds [3]

Dalmatian	Lips
French bulldog	Eyelids, flexure surfaces
German shepherd dog	Elbows, hindlimbs, thorax
Shar-pei	Thorax, flexure surfaces, dorso-lumbar area
West Highland white terrier (WHWT)	Dorso-lumbar area, lips, flexure surfaces
Boxer	Ears

A new tool to assist with the interpretation of the clinical findings when confronted with a pruritic dog is application of clinical criteria known as “Favrot’s criteria” (Table 4) [5]. These include a set of criteria that have been developed from a large case series of confirmed cases of canine AD. The use of complex statistical analysis allowed a set of clinical features to be identified that had maximum association with canine AD. The analysis revealed two sets of criteria, which yield varying levels of sensitivity and specificity for the condition. Clinicians can use whichever set best serves their needs. For example, use of a set of criteria that yields the highest specificity is more likely to ensure that a particular case actually has canine AD. However, this set would exclude some pruritic dogs that were suffering from the disease. A set yielding the highest sensitivity is more likely to capture cases of canine AD, but it could allow some dogs with other conditions to be classified as atopic when in fact they were not. Further guidance about application of these criteria sets is shown in Table 4.

**Table 4:** Favrot’s criteria [5]

	Use	Reliability
Set 1:	• Use for clinical studies and adapt required criteria based on the goal of the study.	• 5 criteria:
1. Age at onset <3 years	• If higher specificity is required, 6 criteria should be fulfilled (e.g., drug trials with potential side effects)	Sens. 85.4 %
2. Mostly indoor	• If higher sensitivity is required, 5 criteria should be fulfilled (e.g., epidemiological studies)	Spec. 79.1 %
3. Corticosteroid-responsive pruritus		
4. Chronic or recurrent yeast infections		• 6 criteria:
5. Affected front feet		Sens. 58.2 %
6. Affected ear pinnae		Spec. 88.5 %
7. Non-affected ear margins		
8. Non-affected dorso-lumbar area		
Set 2:	• Use to evaluate the probability of the diagnosis of canine AD	• 5 criteria:
1. Age at onset < 3 years	• 5 criteria should be fulfilled	Sens. 77.2 %
2. Mostly indoor	• Do not use alone for diagnosis of canine AD, and rule out resembling diseases	Spec. 83 %
3. “Alesional” pruritus at onset		• 6 criteria:
4. Affected front feet		Sens. 42 %
5. Affected ear pinnae		Spec. 93.7 %
6. Non-affected ear margins		
7. Non-affected dorso-lumber area		

It is crucial to remember that these criteria should not be used in isolation as a “diagnostic test” for canine AD. They should be applied alongside the other guidelines outlined in this review. In other words, the accuracy of using these criteria will be greatly enhanced if the dog has been subjected to a careful work-up as described in the previous section.

### Allergy testing

Once a clinical diagnosis of canine AD has been made several factors may play a role in the decision-making whether an allergy test is necessary or not. Severe clinical signs, duration of clinical signs for more than 3 months per year, and insufficient management with symptomatic therapy, due to side effects to the drugs used and/or poor owner compliance, justify in most cases allergy testing. These can be performed by IDT and ASIS. Both tests are not recommended as screening tests and should only be used to confirm the clinical diagnosis of canine AD. The results of these tests are also used to identify the offending allergen(s) in order to formulate an allergen-specific immunotherapy (ASIT). Although IDT is considered the preferred diagnostic method among dermatologists, ASIS has several advantages over IDT, such as: no patient risk (no sedation required), less traumatic (no repeated injection required), more convenient (no clipping needed, less time consuming), and lower risk of drugs interfering with test results (concurrent anti-inflammatory/antipruritic therapy) [45, 46]. However, ASIS only measures circulating allergen-specific IgE, does not take into account other allergic pathways and often shows positive reactions in non-allergic dogs [47, 48].

IDT and ASIS are still lacking standardization and it is suspected that false positive and false negative results do occur. It is estimated that between 10 and 30 % of dogs with a clinically confirmed canine AD may show a negative IDT [49, 50]. This high percentage of false negative results can be due to several factors including improper technique, too low test concentration of allergens [51, 52], drug interference [46], intrinsic host factors, incorrect selection of allergens, IDT performed too long after (>60 days) or during the peak allergy season, and presence of a condition called atopic-like dermatitis [49].

Canine atopic-like disease is clinically identical to canine AD, but IgE response to environmental or other allergens cannot be documented [1]. However, in a recent study the condition has been associated with a lymphocyte-mediated reaction to food [53]. Although it is well known that in people age and season may influence ASIS [54], this information has not been well established in dogs.

Both testing methods are very different and not standardized, which inevitably results in poor correlation between both tests [55]. Nonetheless the success rate of ASIT based on ASIS vs. IDT is not significantly different [56]. Finally, it is important to remember that, although little information is available, cross-reactions between related allergens, e.g., house dust and storage mites, have been reported [57–59]. Based on this problem it is important to determine if a dog is really exposed to the allergen(s) it reacted too. The proper interpretation of these test results, in conjunction with the clinical history and clinical presentation, can be complex and time-consuming. For this reason a referral to a veterinary dermatologist is recommended.

### Intradermal testing

IDT is an indirect measure of cutaneous mast cell reactivity due to the presence of IgE [2]. The appropriate selection of allergens to test is fundamental to obtain reliable IDT results. In fact, allergens, mainly pollens, are subject to a great geographic variability. Thus, it is important for veterinarians performing IDT to identify the allergens present in the regional location where the patients live. Information about relevant allergens can be obtained by contacting veterinary dermatologists, veterinary and medical schools, allergy laboratories, textbooks, local human allergists, weather bureau as well as National Allergy Bureau (http://www.worldallergy.org/pollen/) [49]. From time to time the overall IDT results should be assessed and allergens, which do not exhibit a reaction may be replaced with other important allergens [49]. Intradermal test concentration may also be adjusted since different test concentrations have been suggested over time (Table 5) [49, 51, 52, 60].

**Table 5:** Recommended IDT concentrations for most allergen suppliers

Allergen	Recommended allergen dilution for IDT [49]	Revised recommended allergen^a^ dilution for IDT [51, 52, 60]
Histamine	1: 100,000 w/v	1:10,000 w/v
Pollens and moulds	1,000 PNU/mL	1000 to 8000 PNU/mL
Individual DM	250 PNU/mL or 1:50,000 w/v	100–200 PNU/mL (*D. pteronyssinus*)
		75 PNU/mL (*D. farina, Tyrophagus putrescentiae,* and *Lepidoglyphus destructor*)
		50 PNU/mL (*Acarus siro* and *Blomia tropicalis*)
Epidermal extracts	250–500 PNU/mL	At least 1,250 PNU/mL
		300 PNU/mL (human dander)
Insects	1,000 PNU/mL	At least 1,750 PNU/mL
Whole flea extract	1:1,000 w/v	1:500 w/v

*PNU* Protein Nitrogen Units, *w/v* weight to volume, *DM* dust mites, *D* Dermatophagoides, Epidermal extracts: hair, wool, feathers, and dander

^a^Allergens from Greer Laboratories Inc., Lenoir, NC, USA

Allergens are relatively stable once diluted and can be stored in glass vials up to 8 weeks and in plastic syringes for up to 2 weeks at 4 °C [49]. The test solutions should be removed from the refrigerator just prior to the IDT long enough to reach room temperature. As mentioned before the selection of test allergens should be made based on the prevalence of the allergens in a specific geographical region. However, the selection of test allergens is often based on personal preference and experience and can vary significantly among dermatologists even within the same geographical region [61].

Intradermal injections for IDT are most commonly performed on the lateral thorax, after the hair has been gently clipped and the injection sites marked (minimum 2 cm apart). Typically a volume of 0.05–0.1 ml of each test concentration is injected intradermally and evaluated after 15–20 min. The reaction at each injection site will be compared between those of the positive (histamine phosphate) and negative (saline with phenol) controls. The reaction can be read subjectively and/or objectively. In the first case, assessment of the intensity and/or size of the erythema, turgidity and/or wheal formation will be considered, while for the objective evaluation, measurement of mean diameter of the area of erythema or wheal formation is measured. However, no significant differences were seen where the two methodologies have been compared with each other [62]. By convention, an allergen reaction is positive when the wheal formed is at least equal or greater than halfway between the negative and the positive control reaction. If the subjective evaluation is used, the positive control will assume a conventional grade of 4, whereas the negative control will be graded as 0. A reaction to an allergen is considered positive if it’s graded as 2 or greater [49].

Many positive controls have been tested for IDT in dogs; of those the most reliable is histamine phosphate. Histamine has been used at 1:10,000 w/v (0.1 mg/mL) in Europe and 1:100,000 w/v (0.01 mg/mL) in the USA; nevertheless it has been suggested that the more concentrated solution (1:10,000) may yield a more consistent positive skin reaction [51, 63]. The negative control should consist of the solution, which is used to dilute the allergens for the IDT; this is generally sterile saline with phenol as preservative.

### Allergen-specific IgE serology testing

Several assays, mostly based on solid phase ELISAs, have been tested for serum IgE in both human and veterinary medicine. These assays are used to detect specific IgE antibodies against a panel of allergens (e.g., pollen, mould, HDM and epidermal allergens) considered relevant for the patient. In the past decades, the detection of serum IgE has been done using monoclonal, mixed monoclonal or polyclonal anti-canine IgE. However, due to the higher sensitivity and specificity of a monoclonal antibody, the use of polyclonal anti-canine IgE antibodies has decreased markedly [64, 65]. Another veterinary assay using a unique recombinant fragment of the extracellular portion of the human high affinity IgE receptor alpha-subunit (FcεRIα) has shown a strong affinity for canine IgE and a lack of cross-reactivity with IgG [66, 67]. Two versions of in-clinic immunodot assay, Allercept E-screen^©^ (Heska Corp, Ft Collins, CO, USA) has been validated to detect allergen-specific IgE in canine sera [68, 69]. This test has been used as screening test to guide the veterinarian to determine the possibility to perform a full panel ASIS or IDT using mixtures of flea, HDM and pollen allergens. The Allercept E-screen^©^ immunodot assay was able to predict with high probability whether an IDT and/or ASIS would be negative or positive [68]. However, this test is a screening test using mixed allergen, which does not allow the identification of the individual offending allergen, and so does not replace complete IDT or ASIS testing. Currently many other companies are offering allergen-specific serology testing, but based on a recent study test results do not agree well between laboratories [70].

### Are IDT and ASIS reliable for the identification of canine adverse food reactions?

Many laboratories offer food allergen-specific IgE panels despite the fact that several studies have suggested that IDT and ASIS are not reliable in diagnosing CAFR [49, 71–73]. IDT for example has a very low sensitivity (10–33 %) and a high variable specificity (50–95 %) [49]. Thus, it is worth to reinforce the concept that IDT and ASIS should not be used to make a diagnosis of CAFR.

Some promising results were obtained by patch testing for food components [74], but at this point the test method is at an experimental stage and will require further evaluation.

### Do any drugs interfere with IDT and/or ASIS?

The administration of drugs that can inhibit the release of histamine, and possibly other inflammatory mediators, inducing false negative results needs to be carefully considered when performing an IDT. In fact, antihistamines, glucocorticoids, progestational compounds, β2 adrenergic agonists, bronchodilators, tricyclic antidepressants may interfere with IDT [49]. On the contrary, ketoconazole, essential fatty acids, cyclosporine and oclacitinib seem to interfere less with IDT [75–78]. Similarly, some sedatives should not be used to tranquillize the patient, such as oxymorphone, ketamine/diazepam, acepromazine and morphine [79]. On the contrary, xylazine hydrochloride, medetomidine (dexmedetomidine), tiletamine/zolazepam, thiamylal, halothane, isofluorane, and methoxyfluorane can be safely used [49]. Recommendations on the use of propofol for IDT are still controversial. In one study propofol reduced the histamine reaction, while in a more recent study in atopic dogs the IDT reactions were enhanced [80, 81].

A recent evidence-based review assessed the withdrawal time for IDT and ASIS of commonly used anti-inflammatory drugs [46]. Although withdrawal times may vary due to duration of treatment, dosage and type of drugs, the following withdrawal times for common anti-inflammatory medication have been suggested [46]:

- IDT: antihistamines (7 days), short-acting oral glucocorticoids (14 days), long-acting injectable glucocorticoids (at least 28 days), topical glucocorticoids (14 days), ciclosporin (probably not needed), pentoxifylline (none)
- ASIS: antihistamines (probably not needed), short-acting oral glucocorticoids (none), long-acting injectable glucocorticoids (<28 days), topical glucocorticoids (none), ciclosporin (none)

## Summary

This review shows that canine AD is a complex disease, which can be often associated with other pruritic diseases. Due to the lack of an accurate commercial allergy test to diagnose canine AD, a clinical diagnosis based on exclusion of other possible pruritic dermatoses and Favrot’s criteria is required. Since CARF is often indistinguishable from canine AD properly performed elimination diet trials are required whenever there is perennial pruritus and/or concurrent gastrointestinal signs. Allergy tests should only be used once a clinical diagnosis of canine AD has been made with the primary purpose being to identify potential causative allergens that may be avoided or treated with ASIT. More research is needed to further assess phenotypical variations of canine AD among other breeds, evaluate allergens involving certain body sites, and improve testing methods.

## Abbreviations

AD: Atopic dermatitis
ICADA: International Committee for Allergic Diseases in Animals
IDT: Intradermal Testing
ASIS: Allergen-specific IgE Serology
FAD: Flea allergy dermatitis
CAFR: Cutaneous adverse food reaction
ASIT: Allergen-specific immunotherapy
